# Designing physical activity interventions for women aged 50+: a qualitative study of participant perspectives

**DOI:** 10.1186/s12889-022-14237-y

**Published:** 2022-10-04

**Authors:** Geraldine Wallbank, Abby Haynes, Anne Tiedemann, Catherine Sherrington, Anne C. Grunseit

**Affiliations:** 1 Institute for Musculoskeletal Health, Faculty of Medicine and Health, The University of Sydney and Sydney Local Health District, Sydney, Australia; 2 Sydney School of Public Health, Faculty of Medicine and Health, The University of Sydney, Sydney, Australia; 3 Prevention Research Collaboration, Sydney School of Public Health, The University of Sydney, Sydney, Australia; 4 Missenden Road, PO Box M179, 2050 Camperdown, NSW Australia

**Keywords:** Middle-age, Women, Physical activity, Intervention trial, Qualitative methods

## Abstract

**Background:**

The *Active Women over 50* trial tested a scalable program for increasing physical activity among women aged 50+. The program included information, activity tracker and email support. This study sought to describe the participant perspectives of the *Active Women over 50* program and considerations for designing physical activity interventions for this demographic.

**Methods:**

Women who completed the *Active Women over 50* trial were purposively recruited for maximum variation in age, employment, carer responsibility, medical conditions and physical activity. Individual semi-structured interviews explored their perspectives on physical activity, *Active Women over 50* program components and suggestions for future iterations. Data were thematically analysed.

**Results:**

Participants’ capacity to be physically active was shaped by an interplay of factors. Our analysis generated four main themes relating to physical activity in general and to the program: Age and gender matters, Physical activity is social, Strategising for physical activity and the Self-responsibility discourse. At this midlife stage, physical activity participation was challenged by personal, life-stage and cultural factors, alongside a tension of the self-responsibility discourse which also impacted the program experience. Social factors and finding a suitable strategy for motivation were deemed integral aspects of being active. Future programs could consider facilitation of social networks and accountability, life-stage health information and positive framing to support self-responsibility.

**Conclusion:**

A range of strategies is key to supporting women over 50 to be more physically active due to the variety of circumstances and levels of agency experienced. We offer suggestions that do not need to be resource intensive but could be incorporated into a scaled program.

## BACKGROUND

Physical inactivity is a pressing global public health problem [1–3], accounting for 5.3 million deaths annually and costing health systems an estimated INT$67.5 billion [4]. One in four adults worldwide are physically inactive and for women this proportion increases to one in three [5]. Regular physical activity commenced in middle age years is associated with longevity benefits [6, 7] and can delay physical disability by up to 15 years in women [2]. Physical activity programs that target inactive people, and specifically middle-aged women, not only ameliorate the risks of inactivity but can promote healthy and independent ageing.

Physical activity programs need to be effective and appealing. While effective physical activity interventions have been reported for middle-aged and older populations [8–10], intensive resources needed can be a barrier to implementation at scale [11]. Programs also need to be compelling and address unique barriers to regular physical activity for women over 50, such as juggling carer and work responsibilities [12, 13], or low confidence with being active particularly if they were inactive in younger years [14].

Researchers at the Institute for Musculoskeletal Health, Sydney, Australia, conducted the *Active Women over 50* trial in response to the need for a scalable solution for physical activity[15, 16] *Active Women over 50* was underpinned by behaviour change theory and tested the effect of a resource-efficient, “low-dose” information and support intervention on physical activity in women aged 50 + in the workplace setting[17].

### The Active Women over 50 trial

In brief, *Active Women over 50* trial aimed to increase physical activity participation among female university or local health service employees, aged 50+, English speaking and physically inactive by self-report. One hundred and twenty-six trial participants were recruited and randomly allocated to the intervention group or to a 3-month waitlist control. The intervention was based on behaviour change principles, informed by the Theoretical Domains Framework and the Capability, Opportunity, Motivation, Behaviour (COM-B) framework [18]. Intervention was: (1) a face-to-face information session held at the workplace discussing physical activity benefits, overcoming barriers and strategies for being more active, and viewing peer video interviews. The intervention provided access to (2) a study-loaned *Fitbit* activity tracker; (3) physical activity resource book; (4) fortnightly motivational-based email messages; (5) online *Yammer* platform attendee discussion group; (6) workplace sports facility free trial. Intervention participants planned and chose their preferred physical activity type, amount and strategies. Control participants received the intervention after the follow-up assessment.

Compared to controls at follow-up, the intervention group reported significantly more vigorous-intensity weekly physical activity (1.04 h, 95%CI 0.24 to 1.85, p = 0.012 measured by *International Physical Activity Questionnaire* [19]) and were more likely to achieve upper limits of the World Health Organization physical activity guidelines for moderate-vigorous physical activity (300 min per week [20]) (OR1.98, 95%CI 0.89 to 4.36, p = 0.093). There was no significant between-group difference on the primary outcome of taking 10,000 daily steps (1.39, 95%CI 0.61 to 3.18, p = 0.438).

It was considered essential to explore participants’ perspectives and contexts during trial evaluation to inform future iterations. Previous qualitative studies have described physical activity barriers and facilitators for middle-aged women [21–23] and participant experiences of physical activity programs [24, 25]. Yet, there is a need for qualitative work that synthesises the broader factors shaping physical activity with participant experiences of a physical activity program to better understand the population and guide program design.

The overall aim of this study was to explore factors to consider when designing a physical activity intervention for women aged 50+. Specific research questions were:

1. What were participants’ experiences of physical activity in general?
2. What were participants’ experiences of the *Active Women over 50* program?
3. What are the implications for future iterations of this and other physical activity programs targeting this population?

## METHODS

### Study design and context

We used a qualitative design through semi-structured one-on-one interviews, taking a pragmatist-oriented approach. This approach is atheoretical, socially situated and generates applied knowledge to address the study aims, research questions and adequate action to address the problem [26]. This research study informed the evaluation of the *Active Women over 50*trial[17]. Ethical approval was granted by the Ethics Review Committee at the Sydney Local Health District Research Ethics and Governance Office (X17-0316 & LNR/17/RPAH/473).

### Recruitment and data collection

Women who had completed the *Active Women over 50* trial from both the intervention and waitlist control groups were purposively recruited and invited to participate in this study. Participants were targeted for maximum variation in age, hours worked, work area, carer responsibility, medical conditions and pre-trial physical activity. Potential participants were invited via email during November 2018-January 2019 and interviews were conducted at least three months after the women had completed the *Active Women over 50* trial when waitlist control participants had received the intervention.

Recruitment was a staged process where additional participants were invited to participate following lack of response or declined invitations from earlier rounds of invitations. This process was concurrent with data collection and early analysis and we stopped at the point when data adequacy had been reached i.e., we judged during our analysis that we had sufficient rich data across our sample with which to answer our research questions [27, 28]. Of the 61 women contacted, 18 women (30%) declined the invitation and 23 (38%) did not reply. Twenty women (33%) provided written or verbal consent to participate in the study. GW and AH conducted face-to-face semi-structured interviews at the university research centre, the participant’s workplace, or by telephone according to participant preference. Only the interviewer and participant were present at the interviews. No repeat interviews were conducted.

GW was a PhD candidate with a physiotherapy background, the study manager for the *Active Women over 50* trial[17], and had training in interview techniques and qualitative research. While GW had attended some of the workshops in the trial, to minimise the potential impact this may have had on participants’ perspectives, GW only interviewed participants who attended workshops where GW was not in attendance. AH was an experienced qualitative researcher with a background in social work and program evaluation and had not been involved in the *Active Women over 50* trial. To minimise the potential impact that GW may have had with participants, AH interviewed participants from the workshops where GW was in attendance. GW and AH were aged in their 40 and 50 s and had not previously collaborated.

GW and AH generated the interview guide in consultation with the research team, informed by interpersonal-level program theory such as Social Cognitive Theory. This theory emphasises the dynamic interaction between people, an individual’s prior behaviour and the social and physical environment in influencing future behaviour [29, 30]. Participants were probed on their perspectives of physical activity in general, aspects of the trial such as recruitment, program components and levels of support and suggestions for future iterations. GW and AH completed memos after interviews and met regularly to discuss the data, emergent codes and themes and whether more participants needed to be recruited. Recruitment ceased when ‘data adequacy’ was reached,

Interviews took 36 min on average (range 22–60 min). Interview audio recordings were professionally transcribed and transcripts were corrected by the interviewer. Transcripts were not returned to participants.

### Data analysis

Data were coded by GW using an inductive analytical approach with codes relevant to understanding the participant perspectives and experiences as described by Braun, Clarke and Rance[31]. The initial codes were iteratively grouped into higher level headings by one researcher (GW) and tested for coherence and meaning given the research questions. Coding was discussed with two researchers familiar with the data (AH, AG) early in the analysis for how well the codes captured the participant perspectives and agreement on the coding scheme achieved. One researcher (GW) coded the remainder of the data. Recurrent themes and subthemes were generated from the raw data and were considered in consultation with the wider research team to increase dependability [32]. Themes and subthemes were iteratively reviewed to ensure they reflected the whole dataset, and interpretations were refined. We used [33] software to manage the data. The criteria for reporting qualitative research (COREQ) was used as a guideline[34].

## RESULTS

### Participant characteristics

All twenty participants were female employees at the university or health service, as per trial eligibility. Participant characteristics are summarised in Table 1. Two were colleagues and four worked in the same department. Participants had a median age of 56 years (IQR 53.8 to 58.0) and worked 35 h per week (IQR 28.0 to 40.0) in the fields of administration, architecture, design and planning, arts and social sciences, business and finance, human resources, library, medicine and allied health, offices of General Counsel and the Vice-Chancellor, science and veterinary science. Four participants worked in a health-related field. Nine had carer responsibilities and nine had participated in regular, structured individual or group physical activity when they were younger. Participants spent a median of 348.5 min/week (IQR 202.2 to 477.0) doing moderate-vigorous physical activity measured by accelerometer pre-intervention. We did not find any systematic divergence of the themes based on participant characteristics.

**Table 1:** Participant characteristics

Participant	Age, years	Weekly hours worked	Career type	Carer responsibilities	Regular physical activity when younger (< 50yrs old)	Workshop mode attended	Pre-trial moderate-vigorous physical activity, mins/week
1	57	35	Academic	No	No	In-person	337
2	56	35	Professional, research	No	Yes	In-person	530
3	53	28	Professional, administration	No	No	In-person	126
4	57	35	Professional, research	No	No	In-person	480
5	54	35	Professional, administration	Yes	No	In-person	563
6	51	65	Professional, administration	Yes	Yes	In-person	712
7	60	24	Professional, administration	Yes	No	In-person	487
8	55	28	Professional, administration	No	Yes	Video conference	185
9	56	35	Professional, administration	No	Yes	In-person	187
10	68	40	Academic	No	Yes	In-person	180
11	50	45	Academic	Yes	Yes	Video conference	221
12	58	28	Professional, administration	Yes	No	In-person	360
13	53	50	Academic	Yes	No	In-person	476
14	50	21	Professional, administration	Yes	Yes	In-person	202
15	60	35	Professional, administration	No	Yes	In-person	202
16	56	36	Professional, research	No	No	In-person	380
17	58	35	Professional, administration	No	No	Recording	471
18	54	22	Professional	Yes	Yes	In-person	475
19	57	40	Professional, administration	Yes	No	Recording	319
20	65	40	Professional, administration	No	No	Recording	234

### Main findings

Participants were positive about physical activity. They felt that exercise was good for health and many enjoyed it, and had experienced activity-related physical and psychological benefits. All participants wanted to be more active but felt factors unique to women of their age challenged their ability to enact this desire.

We generated four themes from the narratives which described how participants viewed their capacity to be physically active and how this intersected with the *Active Women over 50* program. These were: (1) “Age and gender matters”, (2) “Physical activity is social”, (3) “Strategising for physical activity”, and (4) “Self-responsibility discourse”. The themes address research questions 1 and 2. Illustrative quotes for each theme are included in Table 2, deidentified to conceal the identity of participants and their workplaces and identified by “P” and participant number only.

**Table 2:** Illustrative quotes for the Themes “Age and gender matters”, “Physical activity is social”, “Strategising for physical activity” and “Self-responsibility discourse” by subtheme and subtheme definition

**Age and gender matters**		
**Subtheme**	**Subtheme description**	*“Particularly in this decade I think where, you know, you’re pressing in from both ends of the generation. You’re caught in the middle, and you’ve still got to try and think about yourself, and your health, and how you’re going to get through.”*(P3)
Life-stage demands	Age and gender-specific demands on life.	
		*“Because I’m over 50 and I’m inactive…but the life changes, menopause happens. I’ve got a very big job, busy as we all are…[exercise was] just a waste of time…I can’t spend an hour going for a walk”*(P13)
Invisibility and Hypervisibility	Perceived a systemic lack of inclusion due to being a woman or ageing woman or both.	“*It’s great if I was 20 years younger, but they [gyms] are not catering for the older person unless they do it [during] work hours, and most older people between 60 and 70 are now working*….*They need to fix something…to show gyms or exercise places that there are older people who need to do it, but they work.”*(P15)
		*“When I first started swimming, actually, I must have had time off and I was a bit overweight and I was shy, embarrassed or shy about my body. So I went to a pool that wasn’t near work. Which was inconvenient …so I didn’t feel as self-conscious.”*(P4).
		*“Often people over 50, in particular, women, are silently ignored and it’s really nice to feel special and that we were worth putting a study together like this.”*(P6)
Bodily changes	Perceived bodily constraints of being a woman or ageing woman or both.	*“I realise the benefits to your health…trying to support my mum and dad…I can see that I’m going to have similar issues if I don’t now do something about it in my earlier years…I don’t want to end up in that situation.”*(P3)
		*“Just life’s just a bit complicated, and…I want to shift that weight again and that 5, 10 kilos that wasn’t there before. Yeah, and now going into my 50s, I’ve got a different metabolism and body than I had when I was younger, so … It’s working with what I’ve got now.”* (P14)
**Physical activity is social**		
**Subtheme**	**Subtheme description**	*“I used to walk with friends and we always just walked and talked. Finding people that you can relate to that you don’t feel you have to be competitive with.”* (P14)
Social connection	Physical activity provided opportunities to interact with others.	
		*“I think just hearing other people’s experience was good… The sort of lifestyles that people have and the way that they can build exercise, whether they’re working or still have family lives… and the way that I could do it. I think that was really motivating.”*(P14)
		*“I remember joining a Yammer [social media] group, and ask if anyone was interested in other people who were walking, and I actually didn’t get any response to that.”*(P10)
Social comparison	Physical activity provided opportunities to appraise oneself against the norm of peers.	*“How are you traveling against other 55 year-olds? Not as a competitive thing, but just for you to kind of go, ‘Okay, I need to up the ante a little bit’, or I need to… You know?… Or ‘I’m doing okay. I’m traveling okay.’”* (P18)
		*“One thing I really liked about the workshop was the videos…For me, that was much more relatable and I was really glad to see that it wasn’t just all these extraordinary woman. It was much more kind of real.”*(P11)
		*“And they [women in video case studies] were highly disciplined people in their jobs. Yeah. So I think [you need] some more realistic type people…”*(P15)
**Strategising for physical activity**		
**Subtheme**	**Subtheme description**	*“Being accountable on a team is a big deal for me, to not let people down, so yeah. Then in my own day-to-day, I don’t feel as accountable to myself. And yet it’s probably the most important thing.”*(P14)
Accountability	Adopting a strategy for being physically active supports the capacity to be active by being accountable to others.	
		*“It was basically left to your own devices. Whether you did what was recommended, there was no one to really put you to the task. …. There was no one to really report back to and say “How are you tracking, how are you going?“…You feel like you’ve achieved something if someone is saying “How are you going? Is it working?“ You know.”*(P3)
Monitoring	Adopting a strategy for being physically active supports the capacity to be active by monitoring one’s own physical activity.	*“It was a good reminder that every time I did a little bit of extra walking it was gonna get recorded. You know? And acknowledged. And it was a good measure of days where things hadn’t gone so well and you go, ’Why didn’t I get my steps in?’”*(P6)
		“…*the following Monday, I come back, and I review what I’ve done, and I can tick off if I’ve managed to do those things, and then I set myself new, kind of, goals, for the next week… I just, kind of, thought, ’Okay. I’ve got to be accountable to myself.’”*(P19)
*Active Women over 50* as a catalyst and resource	Adopting a strategy for being physically active supports the capacity to be active through *Active Women over 50* program components (e.g. motivational email messages, information session).	*“I just never quite got back into that regular exercise pattern and it bothered me. And then when I saw the target group, women 50 or over or something I thought “Okay I should do this.“ Because I’d been casting around looking for an impetus to get me going.”*(P4)
		*“I think I’ve got far more perspective on it all… I also like the fact that the evidence was that it didn’t all have to be hardcore gym…and so that was very good to know that it didn’t have to be costing you a fortune in fees and gear, and what have you…I wanted to be able to not have to rely on other people. Just rely on myself.”* (P6)
		*“Initially it was motivating, but after a while it was obviously, because it’s self-directed, and you’re self-motivating, then yeah. I find that peters out a bit, that self-motivation and encouragement to keep things going.”*(P3)
**Self-responsibility discourse**		
*“It’s so easy to think, and this is my problem, I guess, is that there’s 70 other things that I need to do that will just have to wait. So I guess prioritising, I’m not very good at prioritising.”* (P12)		
*“…, it’s probably more my failure to not use Yammer more and to talk to the other participants online.”* (P12)		
*“The participation in the study was really, really powerful for me…It gave me the opportunity to sit and think and look at this stuff and reassess where I was putting activity and fitness into my priorities and I thought, “No, you’re worth it.“ I want to live long.”* (P6)		

#### Theme 1 – Age and gender matters

A common thread in the narratives was how age and gender shaped physical activity. We identified contextual and personal factors related to age and gender and summarised these under three subthemes: Life-stage demands, Invisibility and hypervisibility, and Bodily changes.

#### Life-stage demands

Life demands which typically came with participants’ age, gender, and stage of career/family life, were often unpredictable and took priority, leaving them with little time for themselves and limited opportunities to be physically active. One such demand was family responsibilities where primary caring duties were typically carried out by women. As members of the *“sandwich generation*” (P19), participants found that inter-generational caring roles necessitated responding to the needs of ageing parents and children, often at the expense of looking after themselves.

Participants described family responsibilities were unpredictable and changeable and since caring duties took priority, physical activity options that required a set time commitment such as gym classes, were not compatible. A similar sentiment was expressed about career-stage demands. Participants reported being of an age where they were working more hours and holding more senior roles which entailed greater responsibility than previously. This included workplace expectations to attend meetings during personal lunchtimes when they could possibly be active.

The demands and unpredictability of family/career responsibilities were so high some participants felt opportunities to be active were not available. Some reported their pattern of physical inactivity persisted when they were no longer in caring roles.

#### Invisibility and hypervisibility

Participants described their willingness to be active was impacted by how they felt their age group and gender were perceived in physical activity settings. They reported that age-specific exercise classes for people aged 50 + at gyms were often scheduled within working hours and made them feel unseen because it assumed their age group did not work.

Participants reported some practices at gyms sent them a message that the *“gym is more for younger people”*(P16). Noting the progressive replacement of gentler class types with high intensity classes, gyms seemed better suited to younger people. Contributing to the suggestion of youth bias in this setting were encounters with gym staff with limited knowledge about age-related bodily changes or age-appropriate exercises. The impression that women aged 50 + were invisible was extended to experiences with other gym patrons. A participant reported others’ assertive use of gym equipment and larger physical build meant *“…you just fade into the background”* (P19), felt ignored or excluded and reluctant to return to the gym. The experience of invisibility in the gym setting as a woman over 50 was expressed as the norm rather than an exception, the contrast which was highlighted by mentions of a participant’s gym being inclusive of older people or the sense of greater visibility and support for being active when attending a women-only gym.

Physical activity also made some participants feel hypervisible and self-conscious about their body shape, fearing it drew unwanted attention and negative reactions. Hypervisibility drove one participant to exercise at a swimming pool in an alternate but inconvenient location.

Participants were discouraged by what they saw as a systemic lack of inclusion of older adults, women, or both, and so tended to reject gyms as a physical activity option. Noting the scarcity of physical activity options suited to women aged 50+, they welcomed the opportunity to contribute to a study that brought greater visibility to their demographic and offered a program to support their physical activity appropriately. This sentiment was not shared by all women aged 50+. A few participants mentioned that the age and gender specific targeting of *Active Women over 50* made their peers feel uncomfortable, hypervisible and *“a bit kind of resistant…[to]the age thing”* (P5), targeting which flagged them as older and drew unwanted attention.

#### Bodily changes

Another factor impacting participants’ physical activity was their perceived bodily constraints. Participants felt they had reached an age where exercise was crucial to their health. Many wanted to “get in early” and be more active to prevent physical decline and maintain independence having witnessed the physical deterioration and dependence of their ageing parents.

However, there was a tension between recognising the need for physical activity to resist decline and feeling the decline had already commenced which made physical activity hard to do. Participants described the awkwardness of having an ageing body which lacked *“resilience”* (P13), energy, experienced disrupted sleep, or gained weight more easily than earlier years. Participants attributed these bodily changes to menopause, being more sedentary, or being older and made it more difficult to be active.

Participants envisaged *Active Women over 50* would help them lose weight and resolve their problem of physical inactivity. But most participants felt the potential benefit of *Active Women over 50* for losing weight was not met conceding that weight loss was difficult at their age and having to *“…work with what I’ve got now”*(P14).

Overall, the theme “Age and gender matters” captured a range of contextual and personal factors that challenged participants’ desire to be more physically active. The age- and gender-specific targeting of *Active Women over 50* seemed to address some of those challenges by recognising women aged over 50 were a group for whom were worth catering.

#### Theme 2 - Physical activity is social

Participants felt that physical activity in general not only benefitted physical health, but it also provided opportunities to interact with other people. In particular, they hoped to be with those who had similar interests which would in turn, provide social support and motivation to be active. We identified two subthemes: Social connection and Social comparison.

#### Social connection

Participants reported physical activity provided opportunities for social interaction and gave a sense of being *“…part of something”*(P18). Participants felt a connection to others gave them the impetus and support to maintain being active, a desire which was felt acutely by a participant who lived alone. However, the mere presence of others was not sufficient to motivate physical activity. Participants cited having mutual interests and similar exercise goals and physical capabilities as providing a supportive social physical activity environment.

Some participants wanted to connect with workplace peers to help support their physical activity but found this difficult due to a predominance of younger employees. Therefore, *Active Women over 50* was valued as an opportunity to meet a “*community*” of “*like-minded*”(P5) women who shared a *“common goal*”(P12) of being active. Participants felt meeting others at a face-to-face workshop at their workplace was particularly appealing. Participants were also attracted to *Active Women over 50* as an age-appropriate and/or family/career-stage-specific modelled solution to being more active within their busy lives.

Participants’ anticipation of connecting with others at the *Active Women over 50* workshop was fulfilled. They described how peer case study videos shown at the workshop prompted attendees to share physical activity ideas and experiences specific to their life-demands. However, many participants reported not feeling connected with other attendees beyond the workshop. Participants reported wanting more opportunities for social connection than *Active Women over 50* offered. One participant explained, the term “workshop” used in recruitment suggested to her the program would facilitate such interactions.

*Active Women over 50* gave attendees access to a private online discussion group via *Yammer* social media to connect beyond the information session. While participants saw the benefits of connecting online with the group conceptually, they reported *Yammer* did not help them because they were neither used the platform, or were comfortable posting comments online. The difficulty of connecting with other attendees through *Yammer* was further described by one participant, whose invitation to shared physical activity time was met with silence. Therefore, an online platform intended to support social connections did not essentially assist participants.

#### Social comparison

Physical activity was social but did not necessarily involve directly interacting with others. Participants were curious to compare themselves against the norm of their peers and reported observing others to appraise their own health as they seemed unsure what was “normal” physical activity for their age. Participants hoped to find a peer group through *Active Women over 50*, feeling it would provide them with a benchmark for making or modifying physical activity plans.

Participants compared their physical activity to peers through viewing the *Active Women over 50* case study videos. Yet, these comparisons seemed to depend on how closely participants identified with the exemplars. Participants who saw they had similar physical activity levels as the exemplars felt the videos were affirming and *“relatable”*(P11). Some who reported they were less active than the exemplars felt the stories were eye-opening and were empowered to be strategic about making firm physical activity plans for themselves. Another participant viewed the exemplars as impressive, but unrealistic and unrelatable comparators.

The theme “physical activity is social” illustrates intrapersonal and interpersonal factors were key in shaping participants’ capacity and motivation to be active. While *Active Women over 50* attracted participants with the anticipation of social supports, the reality fell short of their expectations with too few face-to-face meetings.

#### Theme 3 – Strategising for physical activity

Participants felt it was important to have a strategy for being active to provide structure for accomplishing their goals. Importantly, participants reported the strategy needed to fit with their life-demands and preferences. The theme Strategising for physical activity is conveyed through three subthemes: Accountability, Monitoring, and *Active Women over 50* as a catalyst and resource.

#### Accountability

Overlapping with Theme 2 (“Physical activity is social”) was the concept of accountability. Participants valued social connections through physical activity partly because they wanted to be accountable to other people. They described an implicit commitment when exercising with others that set a “*level of expectation*” (P20) for attendance which they did not have when exercising alone. This sense of obligation validated participants’ efforts and motivated them to keep being active. Yet accountability required shared physical activity abilities and attitudes for it to be supportive. Participants described that a mismatch of these attributes undid their plans to be active with colleagues.

The expectation of accountability was part of the attraction of *Active Women over 50* for some participants. Participants hoped to be *“put to task”*(P9) and be answerable to the program for their physical activity. Program accountability did not come to fruition for these participants who found the program to be “self-directed” and did not recognise their physical activity efforts.

#### Monitoring

Another strategy participants described was monitoring physical activity, via a range of methods such as pedometers, activity trackers, mobile phone applications, or charting activity and goals on a spreadsheet. Through receiving quantified physical activity information participants felt affirmed as their efforts were substantiated, but also caused some *“self-reflection”*(P6) and a motivation to continue being active.

This was also expressed by many who took the opportunity to borrow an *Active Women over 50* pedometer or activity tracker having not using one previously, who felt monitoring their physical activity made them aware of their perceived physical activity levels compared to measured data. A participant reported the fortnightly *Active Women over 50* email messages also acted as a prompt to monitor her physical activity. The messages served as a “*reminder of my intention for going to the workshop originally”* (P4) and cued her to evaluate her physical activity over the past fortnight. Monitoring was therefore a strategy for physical activity that was related to accountability but directed accountability towards the self.

#### ***Active Women over 50*** as a catalyst and resource

For many participants, the program was a tipping point for getting on track with physical activity. They described how the opportune timing of the program at their life stage, conveniently held at their workplace, catalysed them to finally act on ideas they had been contemplating. An important contributing factor was the credibility of the *Active Women over 50* program which participants deemed to be trustworthy, authoritative and non-commercial, attributed to the reputable team and organisation behind it.

Participants described program features of *Active Women over 50* gave them resources for being active. Targeted messages in the workshop and emails about physical activity strategies and options provided awareness, dispelled misconceptions and information to know how to apply it in practice. Despite this, participants reported wanting more program facilitation and external accountability to sustain the motivation they had for being active when they enrolled.

“Strategising for physical activity” involved having a structure for being active. The program came at an opportune time for participants and functioned as a catalyst to be more active. Yet, overall participants wanted more structure than what the program offered to sustain their initial desire to be more active and to provide them with accountability.

#### Theme 4 – Self-responsibility discourse

Participants’ narratives about their physical activity and experience of *Active Women over 50* revealed a strong discourse of personal responsibility. Despite many describing life-demands which were often outside their control, at the same time, participants viewed their inactivity through a lens of self-blame and defeatism. This discourse was typified by sentiments that physical activity *“really is down to me”*(P10) and thus inactivity was considered a personal failure.

In relation to *Active Women over 50*, some participants attributed their failure to be active to poor choices they had made about program features sooner than deficiencies with the program or wider systemic issues. Many felt they would have been more active if they had simply prioritised physical activity or if they had engaged with program emails or the *Yammer* online discussion group. Ultimately, this discourse attributes personal responsibility for success or failure to be active and seemed to mask the role of any broader contributing factors that may have challenged participants’ physical activity. Yet notably, this narrative revealed a sense of agency in some participants who felt participation in the program drove them to reassess their priorities and to take charge of their health and physical activity.

Themes 1 to 4 analysed participants’ experiences with physical activity in general and how these intersected the program. The narratives also identified gaps where *Active Women over 50* could have better supported their physical activity and suggestions for addressing ways the program could be improved in each of the themes (Table 3). Suggestions centred on program initiation and facilitation of additional opportunities to meet other attendees for physical activity and ideas for additional content and delivery options for program features. The suggestions and implications of the findings together address research question 3.

**Table 3:** Summary of participant suggestions for improving *Active Women over 50* against themes

Themes / Subthemes	Participant suggestions for *Active Women over 50* program relevant to theme	Identified gap and recommendation to improve *Active Women over 50*
Age and gender matters / Life-stage demands	*“Maybe some information around menopause and how you cope with that…”* (P2) *“…Except, just like related to having time ‘cause that’s sometimes a big thing. I could go for a 15 minute walk, or I could do something in 15 minutes. Actually, I’ve got an hour, I can probably go to the gym and it won’t be a problem or I’ve only got a quick 30 minute lunch break and I’d like to do something… I’d like to squeeze in something in the evening between making dinner and the washing up or whatever… And maybe things you can do with your family, that involve your family as well.”* (P5)	**Need**: Wider information and solutions for women aged 50+ **Recommend**: Adapt and integrate physical activity information with wider age/gender-related matters. These could include physical activity solutions for people with time constraints or information on broader health topics such as menopause or nutrition.
Age and gender matters / Invisibility and hypervisibility	*[In response to a previous gym experience where staff provided little assistance which was perceived as due to her gender] “I know we had somebody come and talk to us, but it might’ve been nice if we’d gone there, because I’ve never been over to the gym. I’d probably feel a bit intimidated about going for the first time” (P14)*	**Need**: Support attendees’ reticence to use gyms as a physical activity option. **Recommend**: Program facilitation of attendee group visits to the workplace gym and health facility.
Physical activity is social / Social connection	*“I think being able to talk to the participants after the workshop would have been … Yeah, it [would] have been nice just to see what other people’s experience was… Yeah, certainly being able to exchange ideas maybe in a more physical … more sort of face-to-face experience…With the participants and the facilitators, I guess.”*(P14)	**Need**: Opportunities for attendees to share physical activity information/ideas. **Recommend**: Program initiation and facilitation of face-to-face meetings with other attendees beyond the information session.
	*“And I want you [program facilitator] to be there and say, “come on [names self], let’s run around the park today, you’ve got half an hour break, I want to see you out there. Other women are going to be there.“ So I would do more of that. I would do more of that….”* (P9) *“But it might’ve been nice to, you know just thinking in a brainstorm kind of a way, maybe to have been invited to come along to do a lunchtime workout together or something like that. Just to connect doing some kind of physical activity together.”*(P5) *“Not just give you information but actually maybe provide a class so a lot of people can do it and have a feeling and then maybe they’ll start to join.”* (P16)	**Need**: Physical activity opportunities for attendees. **Recommend**: Program initiation and facilitation of face-to-face physical activity opportunities with other attendees beyond the information session.
	*“I think it would be helpful if you actually went out into the community and sort of found groups and shimmied them along…go to their church group or their social group, their book club, their whatever it might be…so you want to build on that emotional requirement.”*(P19)	**Need**: Established social connections to provide support for physical activity. **Recommend**: Leverage established social connections for supporting physical activity by implementing program in community groups.
Strategising for physical activity / Accountability	*“And possibly following up with you [program facilitator] and checking your progress instead of an email, a phone call with a bit of a discussion from the person who does have your baseline, to check in how you’re going, I would have found it very helpful.”* (P6) *“Yeah, that digital thing [pre-intervention accelerometer]. But I never actually found out anything from it. I never actually found out any results from it. So … I guess, I would’ve had a position to, “Okay, well I need to improve on this.“ Yeah.”* (P19)	**Need**: Accountability to someone for being physically active. **Recommend**: Provide accountability to the program via (e.g.) regular “check-in” with attendees; personalisation of program through tailoring, personalised outcomes-focused feedback, and facilitate individual physical activity planning.
	*“Like I said before, if you gave me an exercise physiologist and said okay this exercise physiologist is going to give you a program and then follow you every week or every fortnight or month or whatever, I’d probably be more motivated than I would be, cause I’m scared of it because I now have to live up to his expectation of what I’m meant to be doing. If somebody else is expecting me to do something then I tend more to do it.”* (P20) *“Well, there’s an exercise physiologist who works here at the hospital, but …I don’t have any medically limiting conditions, so really, it’s just motivation I need. So, I don’t need anyone physically … I don’t need anyone to, kind of, show me how to do things. I just need someone behind me, pushing me.”* (P19)	**Need**: Someone to be accountable to for physical activity **Recommend**: Be accountable to a health professional who can provide physical activity personalisation.
Strategising for physical activity / Monitoring	*“So I think something where you can maybe add in your steps, or add in three types of exercise that you’ve done during the week…and that builds into a graph or something… You have a weekly goal. You strive to get to your weekly goal, you get Frequent Flyer points. I have worked out that it’s probably worth, I don’t know, like $30 a year in Frequent Flyer points that you’re getting. So it’s nothing, you know what I mean?”* (P18)	**Need**: Support motivation by linking physical activity to incentives. **Recommend**: Link activity tracker output to a web-based personalised dashboard with set goals. The dashboard could be linked to an incentive program that rewards the attainment of goals.
Strategising for physical activity / *Active Women over 50* as a catalyst and resource	*“And you file it [the email], you categorise it, and then you forget about it…Text? A text might’ve been better. It might’ve been more ding, you know like you kind of get a message and you go, “okay.“… Yeah, a text might be better.”*(P9)	**Need**: Flexibility for receiving messages. **Recommend**: Provide alternate formats other than email to receive motivational messages.
	*“I think videos are always a good thing. Short videos.”* (P5)	**Need**: Engagement with email messages. **Recommend**: Incorporate video content into motivational email messages.

## DISCUSSION

This study explored how participants viewed physical activity in general, the factors that influenced their ability to be active and how these factors intersected with their attraction to and experience of a physical activity information and support program *Active Women over 50*. Our analysis identified four themes: (1) Age and gender matters, (2) Physical activity is social, (3) Strategising for physical activity, and (4) Self-responsibility discourse. Below we contextualise these findings in the broader physical activity promotion literature and draw out the implications for designing appropriate physical activity interventions for women aged 50 and over.

### Contextual factors and tensions with personal responsibility

Our participants experienced individual and systemic level constraints to physical activity which was the context shaping their ability to be active. At an individual level, our findings align with National survey workforce and primary carer data where women comprise 69% of primary carers aged 45–64 years and 10% more women work full-time compared to 20 years ago [12]. Also, the 2018 Australian Institute of Health and Welfare report 62% of women aged 45–64 years live with at least one chronic condition and may experience physiological changes such as (peri)menopausal symptoms or higher prevalence of urinary incontinence [22, 35]. This data suggests that middle-aged women who hold family/carer responsibilities have less leisure time, poorer physical health and enjoyment of physical activity [36, 37].

We found cultural attitudes towards older people had a negative impact on physical activity participation. This has been similarly reported by UK Active case reports [38] which identified systemic barriers for physical activity among older age groups at health facilities. These included inaccessible schedules, an atmosphere of exclusivity, or a workforce lacking sufficient knowledge about age-related health conditions. Other studies have reported women with “fat” body shapes experience explicit negativity when exercising in public places or feeling judged as lazy which meant they avoided exercising publicly [22, 39, 40]. Potentially, a cultural bias towards youth and idealised female bodies may mean the age- and gender-specific targeting of the *Active Women over 50* program could act as a discouragement for some women where the entrenched cultural attitude is perpetuated amongst those whom it biases. For our participants, the program’s specific targeting seemed to address some of those challenges by catering for women over 50. While cultural and systemic factors may influence physical activity, *Active Women over 50* trial outcomes demonstrate that an individual-level program is acceptable and effective among this population.

The strong discourse of self-responsibility was in tension with these cultural and contextual barriers. Despite feeling responsible for physical activity, the time and effort involved for physical activity was often undermined by women’s demanding life circumstances. Waller (2005) explains that despite recognising that these circumstances were often beyond their control, some engaged in self-blame, describing under activity as failure to prioritise or personal inadequacy[41]. Therefore, the personal responsibility discourse motivated women to seek support for physical activity by enrolling in the *Active Women over 50* program but also masked the role of the broader context in following through with being active.

*Active Women over 50* was a behavioural program targeted at an individual level which Adams et al. (2016) reports depends on individual agency to sustain behaviour change[42]. Specific to health, agency is the “individuals’ ability to achieve health goals they value and act as agents of their own health”[43] influenced by broader external conditions[41] . Social and environmental conditions supporting in low individual agency for physical activity are ideal. Yet, our study describes the negative influence that external cultural and systemic attitudes can have on physical activity and together with *Active Women over 50* trial outcomes demonstrating acceptability and effectiveness, an individual-level program among this population is warranted. Clearly, behaviour change is complex and requires a multi-faceted approach at individual and systemic levels.

### The role of social factors

Our findings highlight the role social factors play in motivating physical activity for participants. Holt-Lunstad (2018) defined social connection as connecting with others physically, behaviourally, cognitively, and emotionally and for our participants this conferred a sense of belonging[44]. Participants valued face-to-face opportunities that provided a mechanism for sharing contextualised knowledge about how to be active and for motivation. Participants were also motivated by social factors not requiring interaction but through comparison with others of their age and gender and this reference group was used for self-evaluation [45]. Yet for our participants, the motivation to be active through social comparison was also determined by the extent of identification with the reference group.

Heaney and Israel (2008) describe social support having dimensions of emotional, appraisal, instrumental and informational support which our participants reported were provided by a number of program components[46]. Emotional support was provided through encouragement and reassurance of watching peer video case studies and seeing other “like-minded” women at the workshop; appraisal support via the email messages prompted self-reflection and physical activity planning; instrumental support through loan of the *Fitbit* activity tracker; and lastly, the workshop content provided informational support.

Despite the value of the program components, participants expected and wanted more social opportunities. In particular, participants identified that program-driven face-to-face physical activity sessions and further opportunities to interact with other women of their age would have been more supportive (Table 3). Previous studies describe the unique role that peers play in promoting health behaviours, including program attendance, and emotional, appraisal and informational support which is often based on experiential knowledge [47, 48]. These are compelling reasons to strengthen social factors in future iterations of the program. However, it is noted this study was conducted prior to the COVID-19 pandemic so preferences for face-to-face physical activity interactions may have diminished since. Having adapted to social restrictions, people may be more willing to consider other options, such as via remotely delivered programs.

### Building in a strategy

The *Active Women over 50* program combined a defined program with set components and was designed to give women sufficient flexibly to accommodate their priorities. However, the program was self-driven which disappointed participants who anticipated and wanted some accountability for physical activity – either as a program component or a side-effect of social interaction with peers. ‘External accountability’ and the motivator of “checking in” with research staff was a similar finding reported by Lindgren et al. (2019)[25]. Accountability refers to motivation that arises from the expectation of giving an account and being held responsible for an intention or goal by another person [49].[25] Previous literature suggests that among middle aged populations accountability is heightened by relationship proximity. Within pre-existing social relationships, accountability increased physical activity in both face-to-face and online contexts [50–52]; however between strangers accountability was less likely to increase physical activity [53, 54]. Our study found accountability to health professionals motivated physical activity through their provision of physical activity information, personalisation of physical activity programs, and follow-up reassessment [55, 56]. So, while accountability is a substantial force in motivating behaviour, to whom one is being held accountable has a strong bearing on physical activity.

Another motivating strategy for being active was monitoring also described as “internal accountability” by Lindgren et al. (2019)[25]. The act of observing and recording physical activity seemed to substantiate efforts and provided participants with self-accountability. However, many wanted their physical activity data to be interpreted, evaluated and contextualised against normative values and a peer group through the personal touch of external feedback [25][57]. Among people aged 55 years and over, feedback is a strategy that has been associated with long-term effectiveness of interventions [58] and is a key consideration for future iterations.

Finally, many participants decided to enact their desire to be active and enrol in *Active Women over 50* because of the organisation leading the program. Ranney et al. (2018[59]) and Avery (2010)[60] describe the credibility of an organisation can be integral to whether people heed or ignore health messages. The public evaluate an organisation’s trustworthiness by scrutinising the integrity, competence, motives, public portrayal. Therefore, such trusted organisations need to take advantage of their reach and influence on stimulating and supporting change in health behaviours.

Behaviour change is complex, so to be effective, strategies to tailor an intervention at an individual level need to be addressed. Tailoring an intervention to suit an individual’s circumstances and preferences, underpinned by behaviour change theory and frameworks have been found to be cost-effective [61]. Effective health behaviour interventions could therefore be scalable, that is, be potentially “delivered to an increasing number of participants or through an increasing number of settings, while retaining effectiveness” [15] cited in [16], as a solution for the problem of physical inactivity.

### Implications

Considerations for designing future physical activity interventions to ensure relevance to women aged 50 + are highlighted in this study. First, programs need to adopt an integrative approach to promoting physical activity as a part of overall health and life. By drawing on a range of health and life topics, such as menopause, nutrition and stress management, programs may be able to communicate greater relevance and embed physical activity support strategies within the complex life context described by our participants (Table 3). A broader holistic approach to health may also appeal to a wider variety of people in the target population who may not otherwise consider accessing physical activity support.

Second, the diversity of individual circumstances and motivators for physical activity suggests the need to offer a range of flexible program strategies that appeal to individuals’ situations and preferences, and include solutions that account for time constraints, local resources and physical limitations or disabilities (Table 3). A guided group-visit to the workplace gym, or provision of vouchers to trial facilities could support visibility and attendees’ hesitancy to access unfamiliar physical activity settings. Also, partnerships between program designers and agencies that provide and influence physical activity options (e.g., health facilities, local councils) could help to address barriers to accessing these options.

Third, the benefits of social relationships for physical activity implies the need to facilitate existing or new physical activity social networks (Table 3). Social networks could be leveraged for face-to-face physical activity opportunities, sharing of information and resources and for accountability. Forging new social networks through popular online platforms such as *Facebook* could be feasible. Since adults aged 50 + tend to use *Facebook* for communication [62], the platform could be used to host private discussion groups and physical activity resources such as walking maps and activity logs. The resources required by a program to facilitate a social network among working women in this age group are unknown. Further research is needed to determine for example, the level of input needed from program staff to impact physical activity and studies of cost-effectiveness [48, 63].

Fourth, programs could build in accountability and feedback for physical activity via free government health services such as a telephone health coaching service (e.g. [64] or health/exercise professionals [65] (Table 3). Future iterations could incorporate audit and feedback throughout the program by communicating baseline and periodic physical activity measurements. Tailored feedback could also be provided by digital platforms. A web-based dashboard with an online physical activity diary could track personal trajectories and send messages of praise or suggestions such as “if you’ve set some SMART goals, tell someone”[66].

Fifth, contextual factors and the self-responsibility discourse implies program framing is critical. Establishing the program as a way to support women to combat the pressures of life circumstances and systemic barriers might be a way to engage them in a positive journey of taking responsibility that avoids self-blame. The program needs to adopt motivational, empowering language that explicitly counters the self-blame discourse and recognises the powerful cultural attitudes and practices that make it difficult for women over 50 to be active. Support for realistic goal setting needs to be part of this as failure to meet goals can quickly spiral downwards to a sense of being a failure. Program designers can also work in partnership across sectors that influence physical activity options (e.g., health facilities, local councils) to provide the right conditions for women to be active (Table 3).

Finally, providing the right conditions through systemic change is also important for sustainable behaviour change at a population level, especially given the nuanced interplay of individual, social and wider contextual factors. This is beyond the scope of behavioural programs such as *Active Women over 50*, but it is a reminder that public health researchers have a broader responsibility to advocate for changes in damaging cultural attitudes and practices. In relation to women over 50 and physical activity, our analysis highlights changes in working hours, the division of labour in the home and greater cultural acceptance of ageing women with less-than-perfect bodies. Such long-term and significant societal change can only be addressed by sustained supportive policy, governance and resource allocation across a range of sectors to build a supportive context that encourages people to engage in physical activity[38, 42, 67].

### Strengths and limitations of study

A strength of this study was the exploration of the broader factors facing women over 50 and how these factors shape experiences of a physical activity program. A better understanding of the wider contextual factors for women aged 50 + can guide the design of behavioural interventions to better support this population to be active. A limitation was that while the study drew on a range of participants with differing ages and employment/carer responsibilities as far as the overall sample allowed, study participants were all meeting or exceeding the WHO guidelines for physical activity. Inactive women were not represented in this sample so hearing from those who chose not to participate in the interviews could have provided further perspectives and different views. Another limitation was that one interviewer (GW) had been the study manager of the trial, which may have impacted the willingness of some women to participate, or biased sharing feedback with a trial team member. Other limitations of this study included possible recall bias of the program which was experienced at least 3 months prior and that recruitment occurred during a season where people are typically on summer holidays in Australia (November to January) resulting in a low consent rate.

## Conclusion

Women aged 50 and over face challenges that limit their capacity to be physically active. We identify key challenges described by participants in the *Active Women over 50* program which operate at individual, social and systemic levels. We argue that future physical activity promotion programs targeting this population should offer a range of strategies and structural supports that address different circumstances and levels of agency. We make five suggestions, which do not need to be resource intensive, that could be feasible within a scaled program structure.

## Data Availability

The datasets used and/or analysed during the current study are available from the corresponding author on reasonable request.
